# Diagnostic Yield of Epilepsy Panel Testing in Patients With Seizure Onset Within the First Year of Life

**DOI:** 10.3389/fneur.2019.00988

**Published:** 2019-09-13

**Authors:** Se Song Jang, Soo Yeon Kim, Hunmin Kim, Hee Hwang, Jong Hee Chae, Ki Joong Kim, Jong-Il Kim, Byung Chan Lim

**Affiliations:** ^1^Department of Pediatrics, Seoul National University College of Medicine, Seoul National University Children's Hospital, Seoul, South Korea; ^2^Department of Pediatrics, Seoul National University College of Medicine, Seoul National University Bundang Hospital, Bundang-gu, South Korea; ^3^Department of Biomedical Sciences, Seoul National University Graduate School, Seoul, South Korea; ^4^Department of Biochemistry and Molecular Biology, Seoul National University College of Medicine, Seoul, South Korea; ^5^Medical Research Center, Genomic Medicine Institute, Seoul National University, Seoul, South Korea

**Keywords:** epilepsy, seizure, genetic test, diagnostic yield, target panel sequencing

## Abstract

**Purpose:** We aimed to evaluate the diagnostic yield of epilepsy gene panel testing in epilepsy patients whose seizures began within the first year after birth. We included 112 patients with seizure onset before 12 months and no known etiology.

**Methods:** Deep targeted sequencing with a custom-designed capture probe was performed to ensure the detection of germline or mosaic sequence variants and copy number variations (CNVs).

**Results:** We identified pathogenic or likely pathogenic variants in 53 patients (47.3%, 53/112), including five with pathogenic CNVs. Two putative pathogenic mosaic variants in *SCN8A* and *KCNQ2* were also detected and validated. Those with neonatal onset (61.5%, 16/26) or early infantile onset (50.0%, 29/58) showed higher diagnostic rates than those with late infantile onset (28.5%, 8/28). The diagnostic rate was similar between patients with a specific syndrome (51.9%, 27/52) and those with no recognizable syndrome (43.3%, 26/60).

**Conclusion:** Epilepsy gene panel testing identified a genetic cause in nearly half of the infantile onset epilepsy patients. Since the phenotypic spectrum is expanding and characterizing it at seizure onset is difficult, this group should be prioritized for epilepsy gene panel testing.

## Introduction

With technological advances and declining costs, molecular genetic testing using next-generation sequencing technology is rapidly being incorporated into clinical practice. Although genome-wide testing methods such as whole-exome or whole-genome sequencing are the ultimate goal, selective gene panel tests also have multiple advantages in clinical application (1, 2). Epilepsy gene panel testing is one successful example that has been implemented in clinical practice.

To date, many studies have reported on the clinical utility of epilepsy gene panel testing. Although many custom-designed epilepsy gene panels produce similar lists of genes with pathogenic variants, there is substantial variability in their diagnostic rates, which range from 10 to 50% (3–15). This suggests that the diagnostic yield in these studies depends more on which patients are selected than on which custom-designed panel is used. Previous studies have focused on early-onset epileptic encephalopathy patients, who may be at the severe end of the phenotypic spectrum (4, 12). Recent epilepsy gene panel testing studies have analyzed large numbers of patients with broad epilepsy phenotypes (8, 9, 11, 13, 14). Since most studies report the results of referral-based tests, they include large variability in seizure onset, epilepsy type, familial occurrence, and the presence of development delay or encephalopathy. This variability might lead to lower diagnostic yields, which are generally <20% of the tested patients. One finding common among these studies has been the suggestion that patients with early-onset epilepsy, especially neonatal or early infantile onset, tend to have higher diagnostic rates (8, 9, 11, 14). However, few epilepsy gene panel studies have specifically targeted infantile-onset epilepsy patients and analyzed the diagnostic rate.

Since pathogenic CNVs and somatic mosaicism variants have been reported in a small proportion of epilepsy patients (16, 17), epilepsy gene panel testing capable of identifying these variants would also increase the diagnostic rate. Pathogenic structural variants and low-frequency variants could be readily identified by epilepsy gene panel testing, since targeted testing would ensure deep coverage of a target region.

In the present study, we applied our customized epilepsy gene panel test to a group of epilepsy patients whose seizure onset was before they were 1 year old. We analyzed the diagnostic yield in relation to clinical variables. We also tested the extended applicability of epilepsy gene panel testing by investigating the structural and low-frequency variants in this patient group.

## Materials and Methods

### Patients

The study protocol was approved by the Institutional Review Board of Seoul National University Hospital (1804-052-936), and the study was conducted in accordance with relevant guidelines and regulations. One hundred and twelve epilepsy patients who met the following criteria were included retrospectively: seizure onset before 12 months of age, no structural abnormality on brain magnetic resonance imaging, and no suspected single genetic cause from history and metabolic studies. We only included patients who had initially presented with febrile seizure if they experienced subsequent, afebrile seizures. The following clinical variables were collected: seizure onset, seizure type(s), presence of developmental delay or encephalopathy before and after seizure onset, family history of epilepsy within first-degree relatives, and response to antiepileptic drugs. We tried to classify electroclinical syndromes according to the International League Against Epilepsy proposal (18). Among these patients were 22 with Dravet syndrome, 11 with benign familial infantile epilepsy, 9 with benign infantile epilepsy, 4 with benign familial neonatal epilepsy, 4 with Ohtahara syndrome, and 1 with benign myoclonic epilepsy of infancy. All Dravet syndrome patients had been previously screened with *SCN1A* sequencing and were reported to have no pathogenic variants. We excluded the West syndrome cohort from this study, despite its high prevalence among early-onset epileptic encephalopathy patients, for two reasons. First, whole-exome sequencing studies of large groups of West syndrome patients are already available (19, 20). Second, the West syndrome cohort in our institution was included in another whole-genome-based trio analysis. The detailed clinical features of all 112 patients are summarized in Table S1.

### Epilepsy Panel Design and Sequencing

A custom-designed SureSelect Target Enrichment System Kit (Agilent Technologies, CA, USA) was used to assess epilepsy and epilepsy-associated genes. The capture kits were updated twice to include newly identified genes. Thirty-one patients were sequenced with the first kit (79 genes), 61 with the second kit (119 genes), and 20 (127 genes) with the third kit (Table S2). Library preparation was completed as recommended in the manufacturer's instructions (Agilent Technologies). The library was paired-end sequenced on an Illumina HiSeq 2500 sequencing system.

### Sequence Analysis

We aligned paired-end sequencing reads with a read length of 101 base pairs to Genome Reference Consortium human genome build 37 (patch release 13) using BWA-0.7.15. Picard software (v.2.1.1), SAMtools (v.1.3.1), and the Genome Analysis Toolkit (v.3.8) best-practice pipelines were used for data analyses. Variant calling was performed using HaplotypeCaller. We used ANNOVAR for variant annotation. Using the Exome Aggregation Consortium database, for further analysis we selected only variants with zero frequency in the database for autosomal dominant genes and with a frequency lower than 0.01% for autosomal recessive genes. For low-frequency variant detection, we also used MuTect2 (21) to search for variants with a variant allele frequency from 0.05 to 0.25. We selected only the low-frequency variants with a variant allele count above 30.

For CNV analysis, we calculated reads per kilobase per million mapped reads (RPKM) using CoNIFER (22). Only those reads with mapping quality above 15 were included in the RPKM values. Due to coverage fluctuations among samples in targeted sequencing, we calculated Z-scores twice: within single samples and among multiple samples sequenced in the same panel. With the normalized Z-score values, we calculated the interquartile ranges (IQRs) for each sample. The standards used for identifying CNVs were:

$$\begin{array}{l} \text{ deletion}:\text{Z-score}<\text{q}25-2.5\;\times \text{ IQR}\\ \text{duplication}:\text{Z-score}>\text{q}75+2.5\;\times \text{ IQR} \end{array}$$

where q25 and q75 were the 25th and 75th percentiles of the Z-score values of each sample in each exon. Prominent outlier samples were removed from the analyses for more accurate CNV detection. If more than half of the exons in a gene were amplified or deleted, they were considered for further analysis and testing.

### Variant Interpretation and Validation

All selected sequence variants were further confirmed with Sanger sequencing, which was also conducted for available family members. We classified sequence variants according to the international guidelines of the American College of Medical Genetics (ACMG) (23). Variants classified as “pathogenic” or “likely pathogenic” were considered causative for the phenotype. Low-frequency variants were further validated with amplicon sequencing, in which six nucleotide barcode sequences unique to each sample, along with adaptor sequences (AGAT), were added to forward PCR primer to identify individual samples. Then the same amounts of PCR products for each sample were pooled using an Illumina dual-indexed PCR free library preparation kit and sequenced on an Illumina HiSeq 2500 sequencing system. During sequence analysis, each paired-end read was assigned to an individual by barcode sequences and read numbers, with or without the variant for each sample being counted. To validate CNVs, we conducted chromosomal microarray analysis testing using Agilent Human Genome oligonucleotide comparative genomic hybridization microarrays 4 × 180 K or 8 × 60 K (Agilent Technologies). All experimental procedures and data analyses were performed according to the manufacturer's guidelines (Agilent Technologies).

## Results

The overall coverage of targeted genes was reasonably consistent and deep. The mean coverage depth of 112 patient samples was 1,337 ×, with 98.6% of the target region above 100 ×. A more detailed sequencing summary of all samples in the three different panels is in Table S3. After adjusting the filtering criteria described in the Materials and Methods, ~0–3 single-nucleotide variants were found in each patient. The pathogenic or likely pathogenic variants were found in 53 of 112 patients (47.3%), including five pathogenic CNVs.

### Spectrum of Pathogenic and Likely Pathogenic Variants

#### Sequence Variants

**Figure 1:**
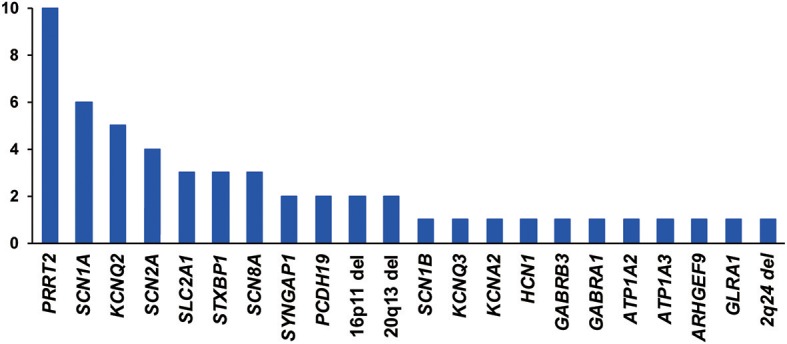
Frequency (y-axis) of genes or copy number variations with pathogenic or likely pathogenic variants.

Eighteen genes were identified as harboring pathogenic or likely pathogenic sequence variants (Figure 1). The most frequently found genes were *PRRT2* in 10 patients, *SCN1A* in 6 patients, *KCNQ2* in 5 patients, and *SCN2A* in 4 patients. Family studies were done in 33 patients. All 6 patients with *SCN1A* pathogenic variants had been previously reported as *SCN1A* mutation negative. This type of missed *SCN1A* mutation has been reported in many studies, indicating the technical limitations of the Sanger sequencing method (24). Seventeen patients were confirmed as harboring *de novo* mutations. Nine variants were inherited from one of the affected parents, and six variants were inherited from one of the asymptomatic parents (Figure S1). We classified these seven variants as pathogenic or likely pathogenic despite the inconsistent familial segregation. The mothers in Case 5 and Case 29 had a mosaic form of the pathogenic variants. Although there is no specific guideline on the interpretation of mosaic variants, asymptomatic parents harboring a mosaic variant of the proband have frequently been interpreted as carriers for the variant (17, 25). The other four variants were either null variants (Case 2, Case 68, Case 78) or a previously reported pathogenic variant (Case 57), which could suggest incomplete penetrance. Table 1 summarizes the pathogenic or likely pathogenic sequence variants.

**Table 1:** Profile of 49 pathogenic or likely pathogenic sequence variants.

**Case**	**Gene**	**Variant (RefSeq:DNA base:amino acid)**	**Inheritance**	**ACMG criteria**	**ACMG classification**	**ClinVar**	**HGMD**
Case 29	*ARHGEF9*	NM_001173479:c.1355G>A:p.Trp452^*^	From mosaic carrier mother	PVS1, PM2, PP1	Pathogenic		
Case 75	*ATP1A2*	NM_000702:c.1096G>T:p.Gly366Cys	*De novo*	PS2, PM1, PM2, PP2, PP3	Pathogenic		
Case 8	*ATP1A3*	NM_152296:c.1088T>C:p. Ile363Thr	Not evaluated	PM1, PM2, PM5, PP2, PP3	Likely Pathogenic		
Case 5	*GABRA1*	NM_001127648:c.1015A>G:p.Lys339Glu	From asymptomatic mosaic mother	PM2, PM6^+^, PP2, PP3	Likely Pathogenic		
Case 66	*GABRB3*	NM_001191320:c.577C>T:p.Leu193Phe	*De novo*	PS2, PM2, PP2, PP3	Likely Pathogenic		
Case 2	*GLRA1*	NM_001292000:c.494_495insAC:p.Met165fs	From asymptomatic father	PVS1, PM2	Likely Pathogenic		
Case 82	*HCN1*	NM_021072:c.1171G>A:p.Gly391Ser	*De novo*	PS2, PM2, PP2, PP3	Likely Pathogenic		
Case 73	*KCNA2*	NM_004974:c.971G>A:p.Ser324Asn	*De Novo*	PS2, PM2, PP2, PP3	Likely Pathogenic		
Case 85	*KCNQ2*	NM_004518:c.727C>G:p.Leu243Val	*De novo*	PS2, PM2, PP2, PP3	Pathogenic		
Case 14	*KCNQ2*	NM_004518:c.766G>T:p.Gly256Trp	*De novo*	PS2, PM1, PM2, PP2, PP3, PP4	Pathogenic		
Case 11	*KCNQ2*	NM_004518:c.997C>T:p.Arg333Trp	Not evaluated	PS2, PS4, PM2, PP2, PP3	Pathogenic	Pathogenic	DM
Case 57	*KCNQ2*	NM_004518:c.998G>A:p.Arg333Gln	From asymptomatic father	PS3, PS4, PM2, PP1, PP2	Pathogenic	Pathogenic	DM
Case 68	*KCNQ2*	NM_004518:c.1130dupC:p.Pro377fs	From asymptomatic father	PVS1, PM2, PP1	Pathogenic		
Case 39	*KCNQ3*	NM_001204824:c.590T>C:p.Ile197Thr	From symptomatic father	PM1, PM2, PP1, PP2, PP3	Likely Pathogenic		DM
Case 56	*PCDH19*	NM_001105243:c.595G>T:p.Glu199^*^	Not evaluated	PVS1, PM2	Likely Pathogenic	Pathogenic	
Case 96	*PCDH19*	NM_001105243:c.1105G>C:p.Ala369Pro	From asymptomatic father	PM2, PP1, PP2, PP3, PP4	Likely Pathogenic		
Case 51	*PRRT2*	NM_001256442:c.649delC:p.Ala217fs	From asymptomatic father	PVS1, PS4, PM1	Pathogenic	Pathogenic	
Case 43	*PRRT2*	NM_001256442:c.649dupC:p.Ala217fs	From symptomatic father	PVS1, PS4, PM1, PP1	Pathogenic	Pathogenic	
Case 58	*PRRT2*	NM_001256442:c.649dupC:p.Ala217fs	Not evaluated	PVS1, PS4, PM1	Pathogenic	Pathogenic	
Case 71	*PRRT2*	NM_0012564c.649dupC:p.Ala217fs	Not evaluated	PVS1, PS4, PM1	Pathogenic	Pathogenic	
Case 77	*PRRT2*	NM_001256442:c.649dupC:p.Ala217fs	Not evaluated	PVS1, PS4, PM1	Pathogenic	Pathogenic	
Case 78	*PRRT2*	NM_001256442:c.649dupC:p.Ala217fs	From asymptomatic father	PVS1, PS4, PM1	Pathogenic	Pathogenic	
Case 81	*PRRT2*	NM_001256442:c.649dupC:p.Ala217fs	From symptomatic mother	PVS1, PS4, PP1	Pathogenic	Pathogenic	
Case 83	*PRRT2*	NM_001256442:c.649dupC:p.Ala217fs	From symptomatic father	PVS1, PS4, PP1	Pathogenic	Pathogenic	
Case 105	*PRRT2*	NM_001256442:c.649dupC:p.Ala217fs	From symptomatic mother	PVS1, PS4, PP1	Pathogenic	Pathogenic	
Case 34	*PRRT2*	NM_001256442:c.796_797insGG:p.Arg266fs	From symptomatic father	PVS1, PS4, PP1	Pathogenic		
Case 26	*SCN1A*	NM_001165963:c.596_602del:p.Thr199fs	Not evaluated	PVS1, PM2, PP4	Pathogenic		
Case 101	*SCN1A*	NM_001165963:c.2244G>A:p.Trp748^*^	Not evaluated	PVS1, PS4, PM2, PP4	Pathogenic		
Case 48	*SCN1A*	NM_001202435:c.2947-1G>A	Not evaluated	PVS1, PM2, PP4	Pathogenic	Likely Pathogenic	
Case 40	*SCN1A*	NM_001165963:c.4201G>C:p.Glu1401Gln	Not evaluated	PM1, PM2, PP2, PP3, PP4	Likely Pathogenic		
Case 13	*SCN1A*	NM_001165963:c.4219C>T:p.Arg1407^*^	Not evaluated	PVS1, PM2, PP4	Pathogenic	Pathogenic	DM
Case 54	*SCN1A*	NM_001165963:c.5288T>A:p.Ile1763Asn	From symptomatic mother	PS4,PM2, PP1, PP2, PP3, PP4	Pathogenic		DM
Case 100	*SCN1B*	NM_001037:c.373C>T:p.Arg125Cys	Not evaluated	PS3, PM2, PP1, PP2, PP3	Pathogenic	Pathogenic	DM
Case 25	*SCN2A*	NM_001040143:c.466A>G:p.Lys156Glu	*De novo*	PS2, PM2, PP2, PP3, PP4	Likely Pathogenic		
Case 45	*SCN2A*	NM_001040143:c.605C>T:p.Ala202Val	*De novo*	PS2, PM2, PP2, PP3	Likely Pathogenic	Uncertain Significance	
Case 33	*SCN2A*	NM_001040143:c.1879C>T:p.Gln627^*^	Not evaluated	PVS1, PM2	Likely Pathogenic		
Case 31	*SCN2A*	NM_001040143:c.2932T>C:p.Phe978Leu	*De novo*	PS2, PM2, PP2, PP3	Likely Pathogenic		
Case 104	*SCN8A*	NM_001177984:c.3820G>A:p.Val1274Met	*De novo*	PS2, PM2, PP2, PP3	Likely Pathogenic		
Case 111	*SCN8A*	NM_014191:c.4423G>A.:p.Gly1475Arg	Not evaluated	PM2, PP2, PP3, PP4, PP5	Likely Pathogenic	Pathogenic/Likely Pathogenic	
Case 79	*SCN8A*	NM_001177984:c.5491C>T:p.Arg1831Trp	*De novo*	PS2, PM2, PP2, PP3	Likely Pathogenic	Pathogenic	DM
Case 47	*SLC2A1*	NM_006516:c.223C>A:p.Gly75Arg	*De novo*	PS2, PM2, PP2, PP3	Likely Pathogenic		
Case 12	*SLC2A1*	NM_006516:c.940G>C:p.Gly314Arg	Inherited from symptomatic mother	PM2, PM5, PP1, PP2, PP3	Likely Pathogenic		
Case 93	*SLC2A1*	NM_006516:c.1255G>C:p.Gly419Arg	*De novo*	PS2, PM2, PP2, PP3	Likely Pathogenic		
Case 17	*STXBP1*	NM_001032221:c.703C>T:p.Arg235^*^	*De novo*	PVS1, PS2, PM2	Pathogenic	Pathogenic	DM
Case 109	*STXBP1*	NM_001032221:c.1099C>T:p.Arg367^*^	Not evaluated	PVS1, PM2	Likely Pathogenic	Pathogenic	DM
Case 64	*STXBP1*	NM_001032221:c.1212A>C:p.Lys404Asn	*De novo*	PS2, PM2, PP2, PP3	Pathogenic		
Case 1	*SYNGAP1*	NM_006772:c.2116-1G>A	*De novo*	PVS1, PS2, PM2	Pathogenic		
Case 36	*SYNGAP1*	NM_006772:c.3718C>T:p.Arg1240^*^	*De novo*	PVS1, PS2, PM2	Pathogenic		

*ACMG, American College of Medical Genetics; DM, Disease causing Mutation; HGMD, Human Gene Mutation Database*;

* *Indicates stopgain*.

#### Structural Variants

We identified five pathogenic CNVs encompassing genes that were included in the present target panel (Table 2). All of these variants were separately validated with chromosomal microarray testing.

**Table 2:** Profile of five pathogenic microdeletions.

**Case**	**Chromosomal position (hg19)**	**Size (Mb)**	**Involved epilepsy genes**	**Onset**	**Electroclinical syndrome**
Case 92	Chr2:165755330-168986256	3.23	*SCN2A, SCN1A, SCN9A*	2 months	Dravet syndrome
Case 16	Chr16:29652999-30198600	0.54	*PRRT2*	4 months	Unclassified
Case 32	Chr16:29673954-30119759	0.44	*PRRT2*	4 days	Benign infantile epilepsy
Case 18	Chr20:61472348-62281707	0.80	*CHRNA4, KCNQ2*	2 months	Unclassified
Case 80	Chr20:61845191-62065069	0.21	*CHRNA4, KCNQ2*	1 day	Benign infantile epilepsy

#### Low-Frequency or Mosaic Variants

Two patients (2/112, 1.8%) were suspected of carrying low-frequency variants in *KCNQ2* and *SCN8A*, respectively (Table 3). The *KCNQ2* and *SCN8A* variants were separately validated with amplicon sequencing. These variants were not found in the parents. Although the *de novo* mosaic status of *KCNQ2* and *SCN8A* variants was demonstrated, we did not classify these variants as pathogenic. The parents of two patients were suspected of having mosaic status for the pathogenic and likely pathogenic variants of *ARHGEF9* and *GABRA1* in the Sanger sequencing results. The *GABRA1* p.Lys339Glu variant was further validated with amplicon sequencing, and it confirmed the mother's mosaic status (Table 3).

**Table 3:** Validation results from amplicon sequencing for the mosaic variants found in patients and parents.

**Case**	**Gene**	**Variant**	**Epilepsy panel sequencing**			**Amplicon sequencing**		
			**References**	**Variant**	**% of variant**	**References**	**Variant**	**% of variant**
Case 61	*KCNQ2*	c.643G>A:p.Gly215Arg	1920	170	8.7%	54190	9933	15.45
Case 61 (Mo)						59508	137	0.23
Case 61 (Fa)						55091	128	0.23
Case 42	*SCN8A*	c.2105G>C:p.Ser702Thr	2427	167	6.4%	19035	4535	19.2
Case 42 (Mo)						15401	129	0.83
Case 42 (Fa)						17973	110	0.61%
Case 5	*GABRA1*	c.1015A>G:p.Lys339Glu	1238	1242	50.1%	59872	53604	47.2%
Case 5 (Mo)						100118	18074	15.3%
Case 5 (Fa)						118255	1269	1.06%

*Fa, father; Mo, mother*.

### Yield by Subgroups

#### Age of Onset

**Figure 2:**
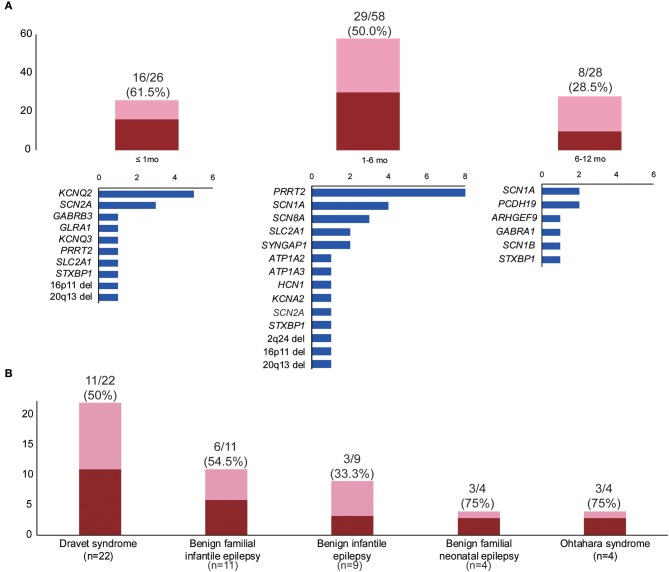
Diagnostic yields by subgroups. **(A)** Diagnostic yields and gene frequencies according to the time of seizure onset: neonatal (≤1 mo), early infantile (1–6 mo), and late infantile (6–12 mo). **(B)** Diagnostic yields according to electroclinical syndrome. The blue bars in **(A)** indicate the number of patients in each group, with the pathogenic or likely pathogenic variants in the genes on the left. The red bar indicates the number of patients with pathogenic or likely pathogenic variants within each group. The pink bar indicates the number of patients without putative variants within each group.

We classified patients into three groups according to age of seizure onset—neonatal, early infantile (1–6 months), and late infantile (6–12 months)—and analyzed the diagnostic yield for each group. The diagnostic yield was higher in the neonatal (61.5%, 16/26) and early infantile (50.0%, 29/58) groups than in the late infantile group (28.5%, 8/28) (Figure 2A). The variants most frequently found in the neonatal group were *KCNQ2* (five patients) and *SCN2A* (three patients), while in the early infantile group, *PRRT2* (nine patients), and *SCN1A* (four patients) were the most frequently found (Figure 2A).

#### Electroclinical Syndromes

The diagnostic rates of patient groups with or without specific electroclinical syndromes were comparable: classified (51.9%, 27/52) vs. unclassified (43.3%, 26/60). The patient group with no specified electroclinical syndromes was more frequently associated with developmental delay or intellectual disability and pharmaco-resistance (Table S4). The diagnostic rate for specific electroclinical syndromes varied and is presented in Figure 2B. In the Dravet syndrome cohort, *SCN1A* sequence variants were found in six patients, although Sanger sequencing performed before panel testing was negative in these patients. The other variants were found in *ARHGEF9* (p.Trp452^*^), *GABRA1* (p.Lys339Glu), *HCN1* (p.Gly391Ser), *PCDH19* (p.Glu199^*^), and 2q24.3 microdeletion. The single *PRRT2* variant (c.649dupC) was found in five patients with benign familial infantile epilepsy. Three of the four benign familial neonatal epilepsy patients revealed pathogenic or likely pathogenic variants in *KCNQ2* (two patients) and *KCNQ3* (one patient). Three of the four Ohtahara syndrome patients showed pathogenic or likely pathogenic variants in *KCNQ2* (p.Gly256Trp), *SCN2A* (p.Lys156Glu), and *STXBP1* (p.Arg235^*^). The remaining Ohtahara syndrome patient also harbored a novel *SCN2A* variant (p.Leu769Thr), classified as a variant of unknown significance due to the absence of a family study.

### Genotype to Phenotype Correlation

Besides the six patients with *SCN1A* variants who could all be classified as having Dravet syndrome, large phenotypic heterogeneity was noted among patients with *PRRT2, KCNQ2*, and *SCN2A* variants. Both severe epileptic encephalopathy and self-limited epilepsies were associated with *KCNQ2* and *SCN2A* variants. Two patients with *PRRT2* variants also showed intellectual disability and behavioral problems that could not be classified as self-limited or benign. The phenotypic spectra of the patients with *PRRT2, KCNQ2*, or *SCN2A* variants are presented in Table 4. In addition to *PRRT2*, which was implicated in both epilepsy and other paroxysmal disorders, we found four variants in genes that cause paroxysmal disorders other than epilepsy: *ATP1A2* (familial hemiplegic migraine, p.Gly366Cys), *ATP1A3* (alternating hemiplegia, p.Ile363Thr), *GLRA1* (hyperekplexia, p.Met165fs), and *ARHGEF9* (hyperekplexia, p.Trp452^*^). These four patients showed varying degrees of developmental delay or intellectual disability and pharmaco-resistance. However, no paroxysmal disorder other than epilepsy was reported in these patients.

**Table 4:** Phenotypic spectrum of patients with *KCNQ2, SCN2A*, or *PRRT2* pathogenic variants.

	***PRRT2* (*n* = 10)**	***KCNQ2* (*n* = 5)**	***SCN2A* (*n* = 4)**
**Epilepsy syndrome**			
	Benign familial infantile (*n* = 5)	Benign familial neonatal (*n* = 2)	Ohtahara syndrome (*n* = 1) Unclassified (*n* = 3)
	Benign infantile (*n* = 3)	Ohtahara syndrome (*n* = 1)	
	Unclassified (*n* = 2)	Unclassified (*n* = 2)	
**Drug responsiveness**			
Self-limited	8	3	1
Drug responsive	2	1	1
Drug resistant		1	2
**Developmental disability**			
Normal	8	3	0
Intellectual disability	1	2	3
ADHD*/ASD*	1	0	1

* *ADHD, attention deficit hyperactivity disorder; ASD, autism spectrum disorder*.

## Discussion

In the present study, a genetic etiology for nearly half of the patients (47.3%) with infantile-onset epilepsy was identified. The higher diagnostic yield in this age group was recently demonstrated in a prospective population-based study by Symonds et al. (26). They prospectively recruited patients whose seizure onset was before 36 months of age. In this study, earlier seizure onset (<6 months) resulted in higher genetic diagnostic yield (45.9%, 34/74) regardless of seizure type and presence of encephalopathy. Thus, these results clearly show the important role of genetic etiology in epilepsy patients with onset in the first year of life.

Infantile-onset epilepsy has several unique features to support the important role of genetic testing. The incidence in this age group is frequently reported to be higher than in all other age groups (27, 28). Moreover, except for West syndrome—in which a structural and metabolic etiology accounts for two-thirds of patients (29)—most of the electroclinical syndromes in infancy had well-characterized genetic profiles as the sole contributing etiological factor. However, we found that patient groups with no recognizable epilepsy syndrome also showed high diagnostic rates (43.3%, 26/60). Thus, age at seizure onset could be the most important factor in genetic diagnosis using epilepsy gene panel testing. We assert that this age-focused approach has an additional advantage over targeting only specific patient groups (e.g., epileptic encephalopathy or drug-resistant epilepsy), insofar as we cannot confidently determine at seizure onset the presence of drug resistance, developmental delay, or encephalopathy. Even self-limited epilepsy syndrome in infancy can only be reliably classified after clinical follow-up beyond infancy. Given that this patient group would benefit greatly from genetic diagnosis at initial presentation to guide treatment and genetic counseling, the age of onset, especially if it is within the first year, should be regarded as the most important indicator for considering genetic testing.

An increasing number of genes are now known to cause both self-limited and severe epilepsies (30). We clearly identified this tendency in the present study, especially for three genes: *SCN2A, KCNQ2*, and *PRRT2*. We expect that an age-focused, unbiased approach to drug resistance and developmental status will reveal this tendency more clearly. Another notable finding regarding the phenotypic spectrum in the present study was that genes related to paroxysmal disorders other than epilepsy could also be associated with epilepsy as a separate phenotype. Benign familial infantile epilepsy is a well-known phenotype of *PRRT2* (OMIM 605751) in addition to paroxysmal dyskinesia (OMIM 128200). *ATP1A2* and *ATP1A3* have previously been implicated in familial hemiplegic migraine (OMIM 602481) and alternating hemiplegia (OMIM 614820). Although the association of infantile epilepsy with these genes has not yet been separately determined, the pathogenic variant in each gene was found in two of our participants, whose epilepsy phenotype could be characterized as developmental epileptic encephalopathy. Additional infantile-onset epilepsy patients linked with *ATP1A2* and *ATP1A3* are found in the literature (8, 9, 15, 31). The independent occurrence of epilepsy and other paroxysmal disorders in a single gene was also reported for *CACNA1A*, an epileptic encephalopathy that has been recognized as a separate phenotype in addition to episodic ataxia and familial hemiplegic migraine (32).

Detection of these five pathogenic CNVs improved the diagnostic rate. All of these CNVs have been reported in infantile-onset epilepsy patients. The phenotypic spectrum in our study was diverse, from self-limited epilepsy to epileptic encephalopathy, even in patients with similar sizes of pathogenic CNVs. Notably, CNV size, which was confirmed by chromosomal microarray, was relatively small, so we could not identify any other genes that may have affected the patient's phenotype other than epilepsy. Since there is no consensus on whether patients with self-limited epilepsy or without dysmorphic features should be tested with a chromosomal microarray, epilepsy gene panel testing could play an important role in identifying epilepsy patients with these phenotypes. In addition to pathogenic CNVs, we found and validated the mosaic variants of *SCN8A* and *KCNQ2* in each patient. Without the mosaic status, these variants might have been interpreted as pathogenic according to the ACMG guidelines. The p.Gly215Arg variant in *KCNQ2* was previously reported in a patient with severe neonatal-onset epilepsy (33). However, we could not confidently classify these variants as pathogenic, since the parents' mosaicism for pathogenic variants in their proband was frequently reported to be asymptomatic (17, 25). Thus, even though the variant could be classified as pathogenic, whether it could result in a clinical phenotype with mosaic status requires separate experimental validation or additional evidence in an unrelated patient. Considering the high frequency of mosaic variants in epilepsy and neuro-developmental disorders (17), more data should be obtained to interpret and validate mosaic variants. Epilepsy gene panel sequencing with deep coverage could be uniquely advantageous for this purpose.

Despite the many advantages discussed above, the limitations of the epilepsy gene panel testing approach need to be addressed. We updated our panel design twice during our study to include newly discovered epilepsy genes. This inevitably leaves a patient group that was not tested for the updated genes. A genome-wide approach, such as whole-exome or whole-genome sequencing, would have a clear advantage over gene panel testing in this situation, because reanalysis could identify additional cases with pathogenic variants in the newly discovered epilepsy genes. However, the benefits and limitations should be weighed carefully based on a head-to-head analysis of cost and diagnostic yield within a specific cohort.

In conclusion, we provided a comprehensive analysis of epilepsy gene panel testing in a group of infantile-onset epilepsy patients, which will contribute to refining the indication of epilepsy gene panel testing by providing a specific test candidate group and expected diagnostic yields.

## Data Availability

We submitted all of the sequenced paired-end reads to the EBI European Nucleotide Archive database with the accession number PRJEB26566 (direct access: https://www.ebi.ac.uk/ena/data/view/PRJEB26566).

## Ethics Statement

The study protocol was approved by the Institutional Review Board of Seoul National University Hospital (IRB No. 1804-052-936), and the study was conducted in accordance with relevant guidelines and regulations. Informed consent was obtained from a parent and/or legal guardian.

## Author Contributions

SJ, BL, J-IK, and JC designed and conceived the study. SK, HK, HH, JC, KK, and BL collected samples, clinical features/data, and ethical statements permitting us to perform the research. SJ and SK analyzed and interpreted the data. SJ and BL reviewed the literature and drafted the manuscript. SK, HK, HH, KK, and JC revised the manuscript for intellectual content.

### Conflict of Interest Statement

The authors declare that the research was conducted in the absence of any commercial or financial relationships that could be construed as a potential conflict of interest.
