# Supplementary figures and images for: Diagnostic Yield of Epilepsy Panel Testing in Patients With Seizure Onset Within the First Year of Life
Source: Front Neurol. 2019 Sep 13;10:988. doi: 10.3389/fneur.2019.00988 (PMC6753218; doi:10.3389/fneur.2019.00988)

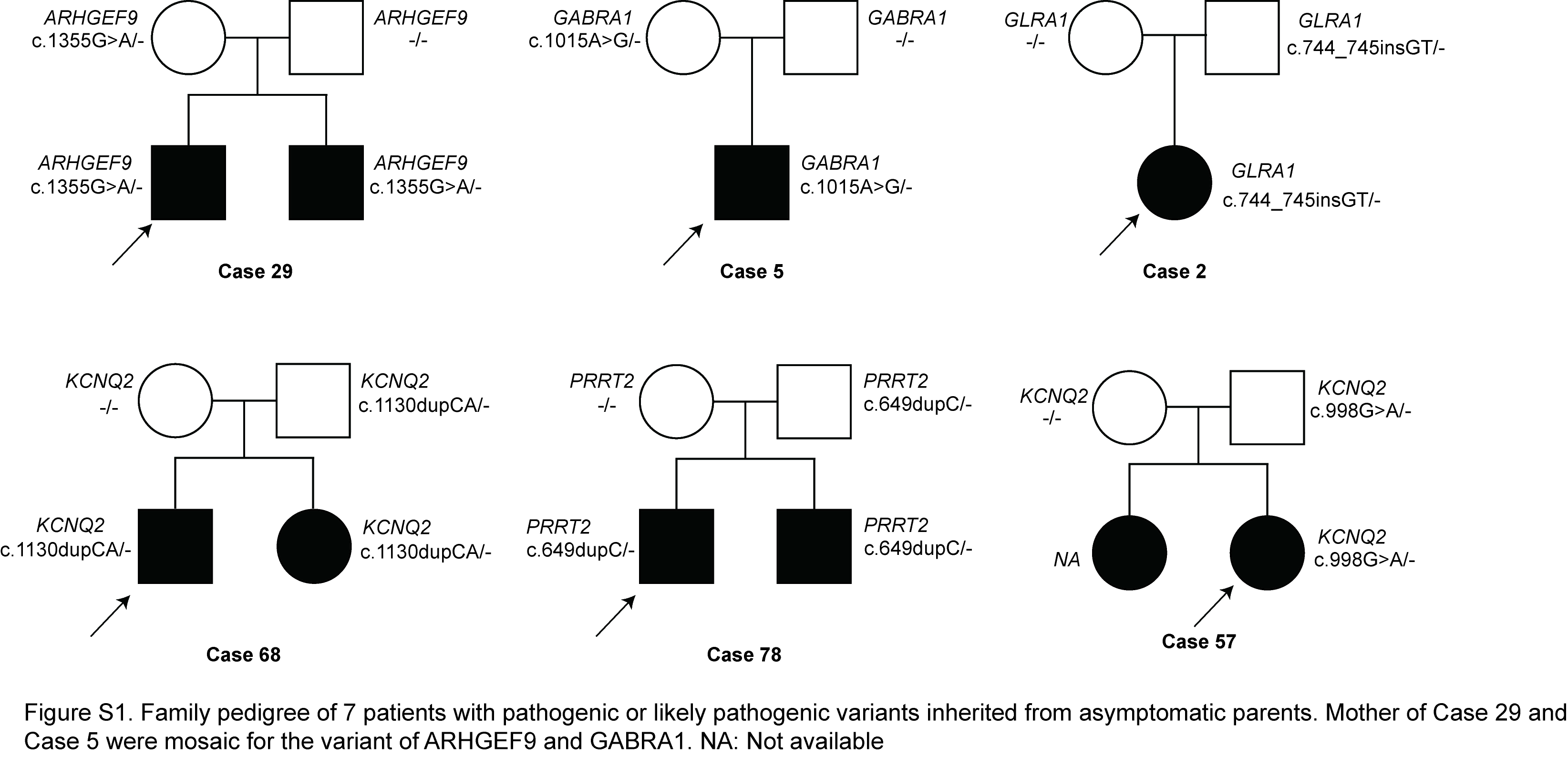

Supplement: Supplementary file 5 [file Image_1.TIF]
